# Novel Application of Multiscale Cross-Approximate Entropy for Assessing Early Changes in the Complexity between Systolic Blood Pressure and ECG R-R Intervals in Diabetic Rats

**DOI:** 10.3390/e24040473

**Published:** 2022-03-29

**Authors:** Wei-Min Liu, Hsin-Ru Liu, Po-Wei Chen, Huai-Ren Chang, Chen-Mao Liao, An-Bang Liu

**Affiliations:** 1 Department of Computer Science and Information Engineering, National Chung Cheng University, Chiayi 621301, Taiwan; wmliu@cs.ccu.edu.tw (W.-M.L.); morris598107@gmail.com (C.-M.L.); 2 Department of Medical Research, Hualien Tzu Chi Hospital, Buddhist Tzu Chi Medical Foundation, Hualien 970473, Taiwan; stylecindy@gmail.com; 3 Medical Department, Hualien Tzu Chi Hospital, Buddhist Tzu Chi Medical Foundation, Hualien 970473, Taiwan; earthqmei@gmail.com; 4 Department of Internal Medicine, Hualien Tzu Chi Hospital, Buddhist Tzu Chi Medical Foundation, Hualien 970473, Taiwan; huairenchang@mail.tcu.edu.tw; 5 School of Medicine, Tzu Chi University, Hualien 970374, Taiwan; 6 Department of Neurology, Hualien Tzu Chi Hospital, Buddhist Tzu Chi Medical Foundation, Hualien 970473, Taiwan

**Keywords:** diabetes mellitus, heart rate variability, baroreflex sensitivity, multiscale cross-approximate entropy

## Abstract

Cardiac autonomic neuropathy (CAN) is a common complication of diabetes mellitus, and can be assessed using heart rate variability (HRV) and the correlations between systolic blood pressure (SBP) and ECG R-R intervals (RRIs), namely baroreflex sensitivity (BRS). In this study, we propose a novel parameter for the nonlinear association between SBP and RRIs based on multiscale cross-approximate entropy (MS-CXApEn). Sixteen male adult Wistar Kyoto rats were equally divided into two groups: streptozotocin-induced diabetes and age-matched controls. RRIs and SBP were acquired in control rats and the diabetic rats at the onset of hyperglycemia and insulin-treated euglycemia to determine HRV by the ratio of low-frequency to high-frequency power (LF/HF) and Poincaré plot as SSR (SD1/SD2), BRS, and MS-CXApEn. SSR and BRS were not significantly different among the three groups. The LF/HF was significantly higher in the hyperglycemic diabetics than those in the controls and euglycemic diabetic rats. MS-CXApEn was higher in the diabetic hyperglycemic rats than the control rats from scales 2 to 10, and approached the values of controls in diabetic euglycemic rats at scales 9 and 10. Conclusions: We propose MS-CXApEn as a novel parameter to quantify the dynamic nonlinear interactions between SBP and RRIs that reveals more apparent changes in early diabetic rats. Furthermore, changes in this parameter were related to correction of hyperglycemia and could be useful for detecting and assessing CAN in early diabetes.

## 1. Introduction

Diabetes mellitus is one of the most common chronic diseases and causes of death in the world [1,2]. Cardiac autonomic neuropathy (CAN) is the most severe complication of diabetes mellitus [3]. CAN not only damages the autonomic nerves innervating the heart, but also affects the blood vessels and circuits between the heart and vessels, and thus impairs homeostasis of the cardiovascular system and leads to complications such as cardiac dysrhythmia, postural hypotension, and hypertension [4,5,6].

Heart rate variability (HRV) is usually used to evaluate CAN. There are several methods to quantify HRV by estimating the variation in ECG R-R intervals (RRIs) in the time and frequency domains. These methods have been applied in clinical studies for many decades. Power spectral analysis assesses the power spectral density of the RRIs by nonparametric methods, e.g., fast Fourier transform (FFT), and parametric methods, e.g., autoregression. The most commonly used frequency bands for humans are the very low frequency (VLF) band from 0 to 0.04 Hz, lower frequency (LF) from 0.04 to 0.15 Hz, and high frequency (HF) from 0.15 to 0.4 Hz [7]. For rats the LF and HF range can be set to 0.20–0.75 Hz and 0.75–3.0 Hz [8]. The frequently assessed parameters include the absolute difference between the powers in LF and HF (|LFP-HFP|), normalized LFP (nLFP), normalized HFP (nHFP), and the ratio of LFP to HFP (LF/HF). The LF/HF has been proposed to represent the modulation of the sympathetic and parasympathetic tones of the heart [9]. The power values in the HF and LF ranges are considered to indicate parasympathetic and sympathetic neural activity, respectively, and the VLF range is believed to be dominated by the sympathetic function [10]. Moreover, HRV can also be evaluated and quantified by a nonlinear method, as the ratio of the standard deviation of the minor axis (SD1) to the standard deviation of the major axis (SD2) from the ellipse of a Poincaré plot of the RRIs, namely SSR [11].

Baroreflex, a rapid negative feedback neural circuit from baroceptors in the aortic arch and carotid sinuses to the solitary nuclei in the brainstem, plays a major role in the control of blood pressure [12]. Elevation of blood pressure activates baroreceptors, then increases parasympathetic activity to decrease heart contractility and slow the heart rate via the vagus nerve, and therefore lower blood pressure [13]. Baroreflex activity has been quantified as baroreflex sensitivity (BRS) based on the correlation between the changes in systolic blood pressure (SBP) and RRIs after intravenous injection of vasoactive agents, the Valsalva maneuver, or spontaneous oscillations [14,15]. Initially, BRS was assessed by pharmacological interventions and has been adopted as a gold-standard method [16]. BRS and HRV assessed by either linear or nonlinear methods have been used to evaluate diabetic CAN in humans and experimental models of diabetes [17,18,19,20,21]. Most clinical or animal studies have investigated the relationships between chronic diabetes and CAN or impaired baroreflex sensitivity [22,23]. However, only a few experiments have addressed autonomic dysfunction in early diabetes [24].

Baroreflex is the temporal association between the change in SBP and relevant RRIs. Since spontaneous oscillation of SBP leads to complex interactions between these two physiological signals, cross-approximate entropy (CXApEn) represents a suitable and useful tool to assess the complex interaction between these two simultaneous signals in the time domain [25,26,27]. In terms of the influence of the microenvironment and interactions between organ systems at different points in time, analysis of the complexity of physiologic systems should not be limited to a single scale. Instead, multiple temporal scales should be considered. Multiscale entropy based on coarse-graining with a weighted summation of scale-dependent entropy can reveal more subtle changes in complexity [28]. Homeostasis within an organism is an integration of dynamic changes in multiple organ systems. Cross-entropy was developed to quantify the synchronicity of two physiological signals in the time domain. These analyses offer useful information to deeply investigate the interactions between two physiological systems in the real world [29,30]. Cross-entropy based on multiscale approaches provides a more sensitive method to study the characteristics of dynamic changes between two physiological signals [31]. We previously used multiscale cross-approximate entropy (MS-CXApEn) to assess the complex interactions between PPIs and the amplitude of digital volume pulse signals acquired by photoplethysmography (PPG) among healthy and diabetic upper-middle-aged subjects. We showed that diabetic patients have significantly lower MS-CXApEns at large scales (scales 4 to 6) [32]. In the current study, we used traditional parameters of the cardiovascular autonomic nervous system, including HRV, BRS, and SSR. As a novel nonlinear method of analysis, MS-CXApEn between SBP and RRIs investigates the early changes in CAN using a rat model of diabetes.

## 2. Materials and Methods

### 2.1. Animals

Adult male Wistar Kyoto (WKY) rats aged approximately 8-weeks-old were purchased from the National Laboratory Animal Breeding and Research Center, Taipei, Taiwan, and housed in a temperature- and humidity-controlled facility maintained at 22–24 °C under a 12 h light-dark cycle, with free access to standard rat chow and water ad libitum, at the Animal Center of Tzu Chi University. All experimental procedures were approved by the Institutional Animal Care and Use Committee of Tzu Chi General Hospital in accordance with the Guide for the Care and Use of Laboratory Animals (IACUC: 102-29), following the guidance of NIH publication no. 85-23, National Academy Press, Washington, DC, USA, revised 1996.

### 2.2. Induction of Diabetes

Eight WKY rats received an intraperitoneal injection of nicotinamide (180 mg/kg, dissolved in 0.9% saline; Sigma, St. Louis, MO, USA) followed by streptozotocin (STZ, Sigma) at a dose of 50 mg/kg 30 min later. The blood sugar levels of the rats were checked using a blood stick, Contour Plus^®^ (Bayer HelathCare, Leverkusen, Nodrhein-Westfalen, Germany) every day. Diabetes was defined as a random sugar level higher than 250 mg/dL [33], which occurred around 5–7 days after induction.

### 2.3. Surgical Procedures and Acquisition of Physiological Signals 

All animals were anesthetized by intraperitoneal injection of urethane (1500 mg/Kg) [34] and placed in the supine position. Arterial cannulation was performed by implanting a PE-50 polyethylene tube in the right femoral artery and the cannula was connected to a pressure transducer (Diagnostic and Research Instruments Co., Ltd., Puzi, Chiayi, Taiwan). Disposable twisted subdermal needle electrodes (SOGEVA S.R.L., Calolziocorte, di Lecco, Italy) were inserted into and fixed on the footpads of the bilateral forelimbs and the tail to record a lead II ECG [35]. 

The left femoral vein was intubated with another PE-50 polyethylene tube for pharmacological intervention. Blood pressure and ECG signals were recorded synchronously by a data acquisition and analysis system, SINGA^®^ Xction View II, Mode XD-04-II (Diagnostic and Research Instruments Co., Ltd.) at a sampling rate of 1000 Hz. These signals were displayed on a monitor to evaluate the baseline stability and changes during the pharmacological intervention. The signals were also recorded and stored in a personal computer for further assessment. We selected a 5-min stable SBP and RRI signals for further analyses of HRV, BRS, and MS-CXApEn. 

### 2.4. Evaluation of Cardiac Autonomic Function in the Frequency Domain

Cardiac autonomic function can be expressed in terms of the spectral features of ECG R-R intervals (RRIs). These features are derived through FFT. The frequency ranges are related to the heart rates and interbeat variations [36]. Unlike the spectral ranges in human HRV, the spectral power fell in ranges: 0.20–0.75 Hz and 0.75–3.0 Hz, denoted as LF and HF, respectively, in assessing HRV in rats [8].

### 2.5. Evaluation of Cardiac Autonomic Function in the Geometrical Domain (Poincaré Plot)

The complexity of RRIs was presented as Poincaré plots generated from consecutive RRI pair series, which are (RRI_1_, RRI_2_), (RRI_2_, RRI_3_), (RRI_3_, RRI_4_), …. The distribution of points usually forms an elliptical-like cluster, and the ratio between the minor axis (SD1) and major axis (SD2) is defined as the SSR (SD1/SD2). The lengths of SD1 and SD2 are derived from the standard deviation of the points in the SD1 and SD2 directions, as shown by the following equations:

(1) $$\mathrm{SD}1=\sqrt{var\left(\frac{RRI_{n}-RRI_{n+1}}{\sqrt{2}}\right)}\;\;\;\;,\;\mathrm{SD}2=\sqrt{var\left(\frac{RRI_{n}+RRI_{n+1}}{\sqrt{2}}\right)}$$

where *var* denotes the variance of the time series.

### 2.6. Assessment of Spontaneous Baroreflex Sensitivity by Sequence Method 

**Figure 1:**
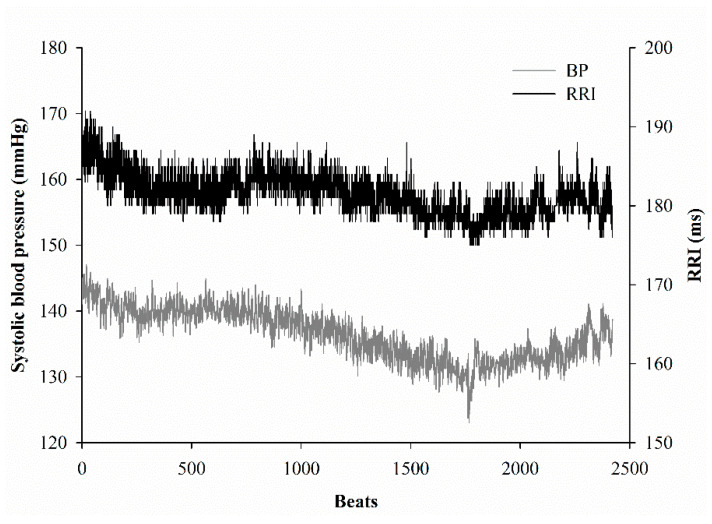
Spontaneous oscillation of systolic blood pressure (SBP, gray line) and R-R intervals (RRI, black line). There is obscure similarity between the changes in SBP and RRI.

**Figure 2:**
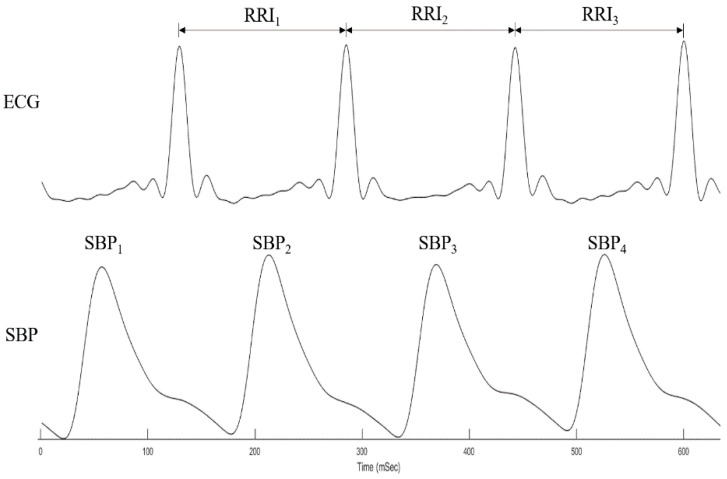
Consecutive systolic blood pressure (SBP_1–4_) and relevant R-R intervals (RRI_1–3_). According to the mechanism of baroreflex, the relevant RRI is defined as the consecutive RRI proceeded to the synchronously recorded SBP.

Figure 1 shows the spontaneous oscillations of SBP and RRIs. We selected a five-minute period of stable SBP and relevant RRIs to assess spontaneous baroreflex sensitivity (BRS_Spn_) by a sequence method and estimate MS-CXApEn. Based on the physiological mechanism of baroreflex, the relevant RRI was defined as the consecutive RRI following the synchronously recorded SBP (Figure 2). The RRI time series were extracted from the ECG after lowpass filtering. The lowpass filter was implemented by an infinite-impulse-response (IIR) filter with a cutoff frequency of 50 Hz to remove AC interference. Such filtering was applied to the SBP signals as well. BRS_Spn_ was defined as the mean correlation between SBP and its corresponding RRI by a sequence method [16]. There were two series: {*SBP*(1), *SBP*(2), …, *SBP*(*N*)} and {*RRI*(1), *RRI*(2), …, *RRI*(*N*)}. We assumed that *n* sets of three consecutive synchronous increases or decreases in SBP(i) and RRI(i) were found. {**SBP(k)**} and {**RRI(k)**} were defined as {SBP(k), SBP(k+1),SBP(k+2)} and {RRI(k), RRI(k+1),RRI(k+2)}. Their means were denoted as $\mu_{\left\{SBP\;\left(k\right)\right\}}$ and $\mu_{\left\{RRI\;\left(k\right)\right\}}$, and the standard deviations were $\sigma_{\left\{SBP\;\left(k\right)\right\}}$ and $\sigma_{\left\{RRI\;\left(k\right)\right\}}$, respectively. The correlation of {SBP(k)} and {RRI(k)} was defined as follows:

(2) $$\;\;\;\;\;\;\;\;\;\;\;\;\;\;R\left(k\right)=\frac{\mathrm{cov}\left(\left\{SBP\left(k\right)\right\},\left\{RRI\left(k\right)\right\}\right)}{\sigma_{\left\{RRI\left(k\right)\right\}}\cdot \;\sigma_{\left\{RRI\left(k\right)\right\}}}\;$$

where cov is the covariance. For each {**SBP(k)**} and {**RRI(k)**} paired set the *R*(*k*) value can be derived, and the slope when considering SBP as the *x*-axis and RRI as the *y*-axis was calculated using the least-squares method [16]. Among the *n* sets, assuming there were *b* sets with an *R* value greater than 0.85 [11], the mean of all slopes was determined as the spontaneous baroreflex sensitivity, BRS_Spn_, using:

(3) $$\;\;\;{\mathrm{BRS}}_{\mathrm{spn}}=\frac{1}{b}\left(\sum_{m=1}^{b}slope\left(m\right)\right){\;}_{\;}$$

### 2.7. Multiscale Cross-Approximate Entropy for the Complexity between Systolic Blood Pressure and ECG R-R Intervals

Cross-approximate entropy was used to explore the complex interaction between two synchronous series. After a coarse-graining process, MS-CXApEn can be used to observe CXApEn under different sizes of observation windows. In this study, we used the same 5-minute stable SBP and RRI signals for MS-CXApEn analysis. Prior to calculating CXApEn, the two series were normalized by subtracting their mean and then divided by their standard deviation. 

Then, CXApEn was calculated in five simplified steps, as follows:

1. Each RRI and SBP time series with length N was chopped into a set of *N* − *m* + 1 vectors. The vectors with length *m* were expressed as:
  $$RRI_{m}^{i}=\left[\mathrm{RRI}\left(i\right)\;\;RRI\left(i+1\right)\;\;\cdots \;\;RRI\left(i+m-1\right)\right]\;\mathrm{and}$$
  $$SBP_{m}^{j}=\left[\mathrm{SBP}\left(j\right)\;\;SBP\left(j+1\right)\;\;\cdots \;\;SBP\left(j+m-1\right)\right],$$
  where *i* and *j* are indexes in the range [1, *N* − *m* + 1].
2. A distance measure that calculates the maximum absolute difference between RRI and SBP vectors was defined as
  (4) $$d\left(SBP_{m}^{j},RRI_{m}^{i}\right)=\underset{1\leq k\leq m}{\max}\left|RRI\left(i+k-1\right)-SBP\left(j+k-1\right)\right|$$
3. A probability can be derived by counting the number of $d\left(SBP_{m}^{j},RRI_{m}^{i}\right)$ that is less than a predefined threshold, *r*
  (5) $$P_{RRI,\;SBP}^{i,m}\left(r\right)=\frac{num\left\{d\left(SBP_{m}^{i},RRI_{m}^{i}\right)<r\right\}}{N-m+1}$$
4. The average logarithm of the probability was defined as
  (6) $$\phi_{RRI,\;SBP}^{m}\left(r\right)=\frac{1}{N-m+1}\sum_{i=1}^{N-m+1}lnP_{RRI,\;SBP}^{i,m}\left(r\right)$$
5. The estimated CXApEn was calculated using $\phi_{RRI,\;SBP}^{m}\left(r\right)-\phi_{RRI,\;SBP}^{m+1}\left(r\right)$.

MS-CXApEn was determined by calculating the CXApEn between RRI and SBP signals under different scales τ; here, τ ranged from 1 to 10. The scale of the RRI signal was defined using Equation (7). The same definition also applied to the SBP signal. Herein, m = 3 and r = 0.25.

(7) $$\mathrm{RRI}{\left(u\right)}^{\left(\tau \right)}=\frac{1}{\tau }\sum_{i=\left(u-1\right)\tau +1}^{u\tau }RRI\left(i\right),\;\;1\leq u\leq \frac{1000}{\tau },u\in N$$

The scales 1–3, 4–6, and 7–10 were considered as small, medium, and large scales, respectively. For the convenience of statistical analysis, the MS-CXApEns for the ten scales were averaged over three scales (1–3, 4–6, 7–10) and defined as MS-CXApEn_small_, MS-CXApEn_medium_, and MS-CXApEn_large_, respectively. 

### 2.8. Surrogate Data Test

To verify that the coordination level between RRI and SBP was significantly different from the interaction between two meaningless signals, we generated two types of surrogates. One was the paired shuffle operation, which applied one set of randomly shuffled order to both RRI and SBP signals. In this case, the one-to-one correspondence between two signals was still maintained. The other is called separated shuffle operation, which generated two independently random shuffled orders, and applied these to RRI and SBP separately. Theoretically, the coordination between the two physiological signals should disappear after the shuffle operation. Such disorganization makes the cross-approximate entropy increase.

### 2.9. Evaluation of Baroreflex Activity by Pharmacological Intervention

After 30-minute acquisition of physiological signals, the animals were intravenously injected with 100 µL of phenylephrine at 8.0 µg/mL (Sigma-Aldrich, St. Louis, MO, USA) via the left femoral vein to induce an increase in SBP by 20 to 40 mmHg above the baseline pressure. The regressions between the changes in SBP (ΔSBP, mmHg) and prolongations of RRIs (ΔRRI, mSec) were defined as BRS_Phe_. A minimum of four consecutive increases in SBP with a coefficient > 0.8 was considered valid [16].

### 2.10. Study Design and Protocols

We used eight WKY rats at an early stage of experimental diabetes and eight age-matched normal rats in this study. Blood sugar levels were checked using Contour Plus^®^ blood sticks after intravenous and intra-arterial cannulation. Then, blood pressure and ECG signals were recorded for 30 min after equilibration. A five-minute period of stable signals was used to assess BRS, FFT, and SSR. Pharmacological intervention of baroreflex activity was performed after the acquisition of baseline physiological signals. The diabetic rats received subcutaneous injection of 100 IU/mL insulin when their blood sugar level was under 400 mg/dL, and received 2 units of injection when the level was higher than 600 mg/dL. Their blood sugar levels were checked an hour after the pharmacological intervention. To investigate the changes in the cardiac autonomic function of the diabetic rats in euglycemic status, blood pressure and ECG signals were also recorded when their blood sugar was less than 200 mg/dL. 

### 2.11. Statistical Analysis

Data are expressed as mean ± standard deviation (SD). The significance of the differences in body weight, blood pressure, heart rate, and the computed parameters of cardiac autonomic function including LFP, HFP, LFPn, HFPn, LF/HF, SSR, BRS_phe_, MS-CXApEns from scales 1 to 10, MS-CXApEn_small_, MS-CXApEn_medium_, and MS-CXApEn_large_ between groups were determined by ANOVA with Fisher’s least significant difference post hoc test. To assess the agreement between normalized MS-CXApEn at different scales and LF/HF, SSR, and BRS_phe_, we used Bland–Altman plots [37] to present the agreement between MS-CXApEn at different scales with other cardiac autonomic parameters. All statistical analyses were performed using STATA software (version 16.0 for Windows; STATA Corp. LLC, College Station, TX, USA). *p*-values < 0.05 were considered statistically significant. 

## 3. Results

### 3.1. Physiological Parameters of Normal Rats, and Diabetic Rats in Hyperglycemic and Euglycemic States

Table 1 compares the essential physiological parameters of the normal rats and the diabetic rats in hyperglycemic and insulin-treated euglycemic states. The rats with experimental diabetes exhibited significant body weight loss compared to their control littermates after the onset of diabetes (303.75 ± 24.83 g vs. 265.25 ± 35.90 g). These diabetic rats also had significantly lower blood pressure and slower heart rates. The cardiovascular parameters approached the values estimated in normal controls after correcting hyperglycemia. However, there was no statistical significance. 

### 3.2. Changes in MS-CXApEns, BRS, Spectral Analysis, and Poincare Plotting of HRV in the Normal Rats and Diabetic Rats in Hyperglycemic and Euglycemic States

**Figure 3:**
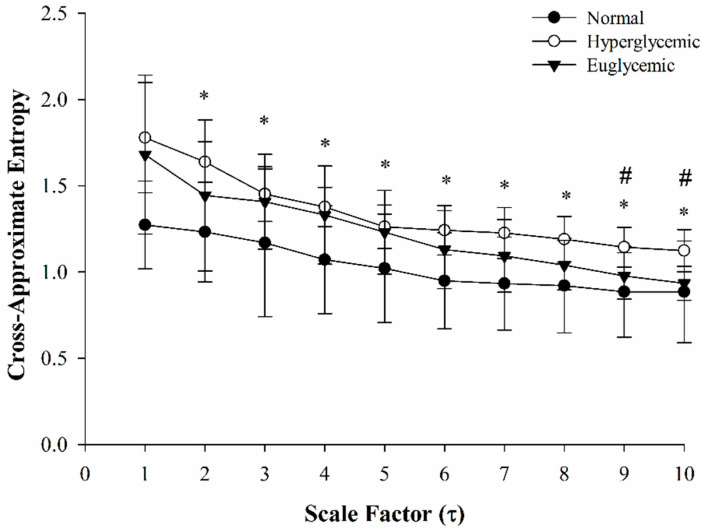
Multiscale cross-approximate entropy between systolic blood pressure and relevant R-R intervals from scales 1 to 10 in the normal controls (black circle), early diabetic rats in hyperglycemic state (white circle), and diabetic rats in euglycemic state (black triangle). *: significant difference between the controls and hyperglycemic rats; #: significant difference between the diabetic rats in hyperglycemic and euglycemic states. Significances of difference were determined as *p* < 0.05 by ANOVA with Fisher’s least significant difference post hoc test.

**Figure 4:**
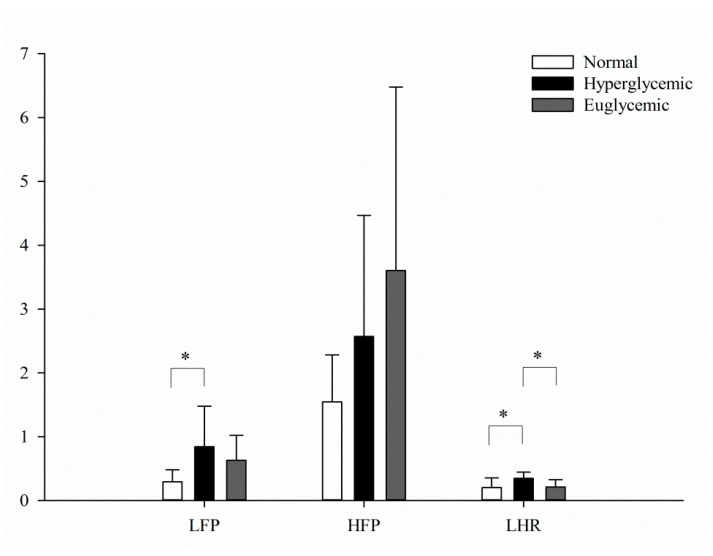
Spectral analyses of HRV among the normal controls (white) and diabetics in hyperglycemic (black) and treated euglycemic states (gray). *: Statistical significances were defined as *p* < 0.05 by ANOVA with Fisher’s least significant difference post hoc test.

Figure 3 demonstrates the changes in MS-CXApEn from scales 1 to 10 in the normal rats, as well as the diabetic rats with early hyperglycemia and euglycemia after injection of insulin. There were significant differences in MC-CXApEn from scales 1 to 10 between the normal controls and rats with hyperglycemia. Significant differences in scales 9 to 10 were also observed between the diabetic rats in hyperglycemic and euglycemic states. As shown in Table 2, there was no significant difference in heart rate variability by Poincaré plot. However, the LFP and LF/HF were significantly higher in the diabetics than those in the normal controls. These two parameters decreased after insulin treatment, but only LF/HF was significantly lower in the euglycemic diabetic rats (Figure 4). There were significant differences in MS-CXApEn on the small-, medium-, and large-scales between the normal control rats and diabetic rats in hyperglycemia. Moreover, MS-CXApEn at scales 9 and 10 was also significantly different between the diabetic rats in hyperglycemic and euglycemic states while classic measures such as SSR and BRS were not among these three groups.

### 3.3. Surrogate Data Test

**Figure 5:**
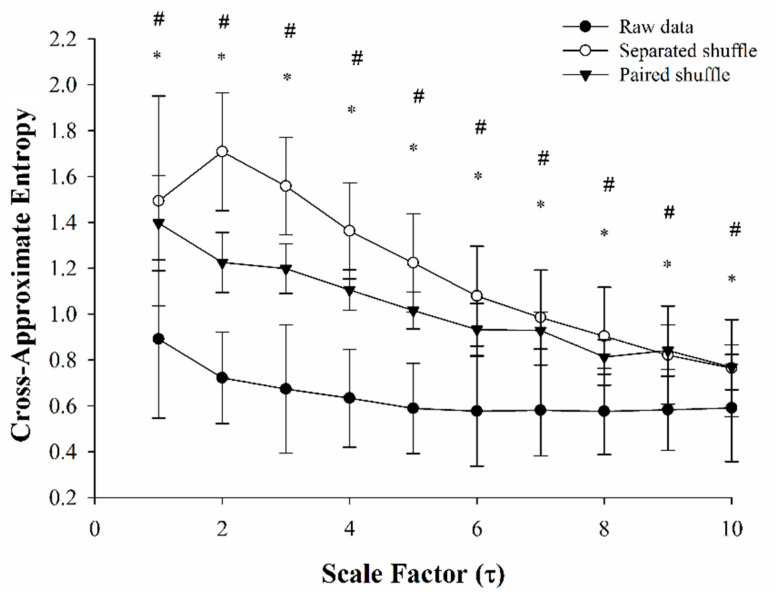
Multiscale cross-approximate entropy of RRI-SBP raw data (black circle), surrogate shuffle series: shuffled paired RRI-SBP (shuffled paired, black triangle), and shuffled RRIs and shuffled SBP (separate shuffled, white circle). *: significant difference between the raw data and paired shuffle series; #: significant difference between the raw data and the separate shuffled series. Significances of difference were determined as *p* < 0.05 by ANOVA with Fisher’s least significant difference post hoc test.

Figure 5 shows that the raw CXApEn values of RRIs and SBP were significantly lower than those in the shuffled paired and separate shuffled RRI and SBP series from scales 1 to 10. 

### 3.4. Agreement between MS-CXApEn at the Small-, Medium-, and Large-Scales and LF/HF, SSR, BRS_spn_, and BRS_phe_ in the Normal Control Rats

**Figure 6:**
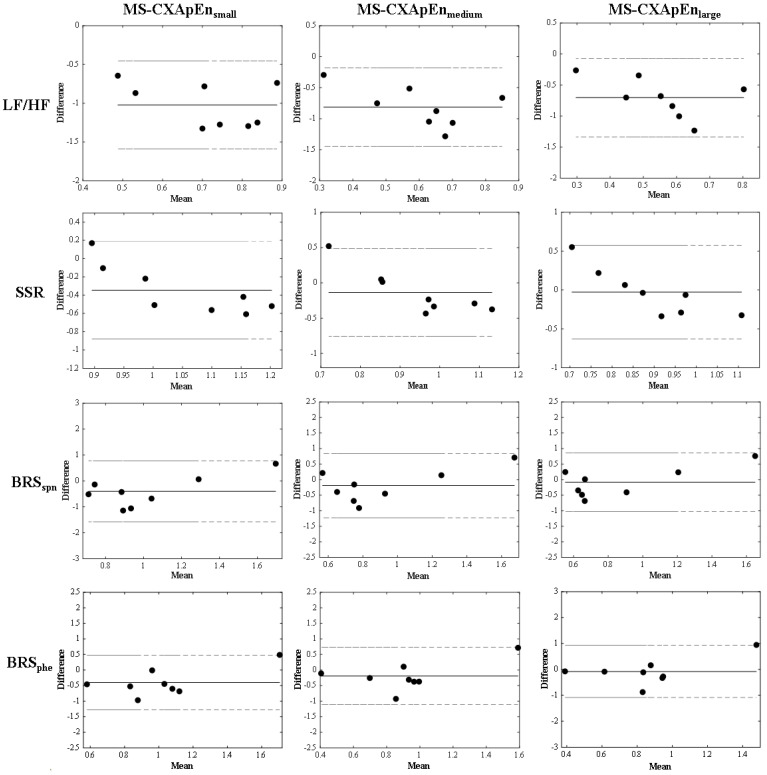
The Bland–Altmann plots presents the 12 sets of agreements between the 3 types of entropy and 4 physiological parameters in the control rats. The 3 types of entropy are multiple scale cross-approximate entropy at small scale (scales 1–3; MS-CXApEn_small_), medium scale (scales 4–6; MS-CXApEn_medium_), and large scale (scales 7–10; MS-CXApEn_large_). The 4 physiological parameters are heart rate variability by fast Fourier transform (LF/HF), Poincaré plotting (SSR), baroreflex sensitivity assessed by sequence method (BRS_spn_) and by pharmacological intervention (BRS_phe_). The agreements are acceptable between the 1.96 SD.

Figure 6 displays the good agreement between MS-CXApEn at all CAN parameters: HRV based on LF/HF and SSR, BRS_spn_, and BRS_phe_ in the normal control rats.

## 4. Discussion

Diabetes mellitus caused by either insulin deficiency or insulin resistance interrupts glucose transport from the blood to the cells. In addition, due to elevated blood sugar levels, bodyweight loss is a frequent presentation of early diabetes. We observed a significant change in blood sugar levels, and the diabetic rats tended to have lower body weight and blood pressure, and slower heart rate at the onset of hyperglycemia compared to the normal rats, though these changes were not significant. Our results are similar to previous studies on early changes in these physiological parameters in rats with STZ-induced diabetes [24,38]. 

The dynamic balance of autonomic nerve function and tones, and changes in intrathoracic pressure during respiration, etc., result in spontaneous oscillation of blood pressure and consequently cause irregularities. Figure 1 shows the oscillations in SBP and RRIs, and their temporal coherence. The sequence method is commonly used and embedded in the commercial device Finapress^®^ to estimate BRS by linear regression of relevant increases or decreases in SBP and RRIs. This approach has been applied in many clinical studies [39]. Moreover, linear regression of the changes in SBP and RRIs after administration of vasoactive agents to increase or decrease SBP has been applied for decades as a gold-standard method of evaluating BRS [16]. However, these linear analyses may not be sensitive to the rapid and minute changes in baroreflex activity under actual physiological situations [40]. Multiscale cross-approximate entropy offers a sensitive method to assess the complex correlations of two physiological signals as a time series. Since CXApEn has been used to estimate the temporal chronicity of two series, we hypothesize that an impaired baroreflex would increase the entropy. MS-CXApEn at scales of 1 to 10 was higher in the diabetic rats than the controls (Figure 3). In contrast, in our previous study of human subjects, MS-CXApEn based on the amplitudes of digital volume pulse and RRIs were significantly higher in healthy subjects than diabetic subjects at scale factors greater than 4 [32]. This may be due to subjects with chronic diabetes probably being affected by concomitant peripheral vascular disease. The current assessment of the interaction between SBP and RRI truly demonstrates that dynamic changes in baroreflex activity occur in early diabetes, in the absence of confounding factors.

To simplify the statistical analysis, we stratified MS-CXApEn into small, medium, and large scales. In addition to assessing baroreflex activity based on the interaction between blood pressure and RRIs, HRV has been used to evaluate the influence of sympathetic and parasympathetic interactions on the heartbeat based on RRIs. Since CAN is a major, severe complication of diabetes, early detection and intervention would be helpful to decrease the related morbidity and mortality rates. HRV was found to be an early indicator of diabetic CAN in a multi-center prospective study [41]. To widen the assessment of autonomic function in early experimental diabetes, we used linear analysis of features from FFT and nonlinear analysis based on Poincaré plotting to evaluate HRV. We also applied the sequence method and pharmacological intervention to evaluate BRS in this study.

The MS-CXApEn values for all scale groups were in good agreement with other assessments of cardiac autonomic function, including LF/HF, SSR, BRS_spn_, and BRS_phe_ in the control group (Figure 6). These findings suggest that the MS-CXApEn values were similar to the cardiovascular autonomic parameters in the control rats. The frequency analysis of HRV shows significantly higher LFP and LF/HF in the diabetic rats as compared with the normal controls. The LFP and LF/HF decreased and HFP increased in the insulin-treated euglycemic diabetic rats, and only LF/HF significantly decreased. There was no significant difference in BRS_phe_ or BRS_spn_ among control rats and diabetic rats (Table 2). However, the MS-CXApEn values at all scale stratifications were significantly higher in the diabetic rats than the control rats. After correcting hyperglycemia, MS-CXApEn in scales 9 and 10 significantly decreased. Nevertheless, the hyperglycemic rats tended to have lower blood pressure and a slower heart rate, but these changes were not significantly different to the control rats or euglycemic diabetic rats (Table 1). These changes in MS-CXApEn may indicate that an impaired baroreflex is the earliest manifestation of diabetic CAN, and consequently results in hypertension. Coexisting hypertension is common in patients with diabetes; however, related longitudinal cohort studies in human subjects are lacking. One study showed that hypertension existed in Wistar rats with chronic, experimentally induced diabetes, and could be restored by supplementation with magnesium sulfate [42].

Costa and colleagues demonstrated that the entropy of white noise and RRIs of atrial fibrillation decreased with scales and increased with the scale factors in healthy subjects [28]. In contrast, the current study shows that cross-approximate entropy decreased with scale, while the normal controls had lower MS-CXApEn than the diabetic rats (Figure 3). To ensure the observation shifted away from the background noise, we performed a surrogate shuffle test. Figure 5 demonstrated that the CXApEn of shuffled signals also decreased with scale factors. Furthermore, the separated shuffled signals had the highest entropy. MS-CXApEn of paired shuffled signals were also higher than those in the normal controls. Unlike multiscale entropy, CXApEn is a sensitive assessment to evaluate the synchrony of two relevant physiological signals such as RRIs and SBP. The MS-CXApEn of two relevant series decreases with scale [43,44,45]. Under the control of baroreflex, the complexity of synchrony between RRIs and SBP decreased with scale factor and CXApEn was lower in the normal controls. The entropy increased while the impairment of baroreflex occurred. We propose that the MS-CXApEn could be a sensitive indicator for early impairment of baroreflex activity in diabetics. The surrogate shuffle test supports this speculation. 

The multiscale entropy allows us to sensitively assess the complexity of a series by including multiple time-scales of measurement after coarse graining. According to this concept, the MS-CXApEn of RRI and SBP at small scales may correspond to low-frequency signals, namely respiration and sympathetic activity. In the meantime, the MS-CXApEn in large scales probably relates to the parasympathetic tone. Interestingly, Figure 3 shows that the diabetic rats in hyperglycemic states had higher CXApEn from scales 1 to 10 as compared with the normal controls. The significant difference in CXApEn only existed at scales 9 and 10 between hyperglycemic and euglycemic diabetics. Table 2 also shows significant differences in LFP between the hyperglycemic diabetics and normal controls. It demonstrates the tendency of increments of HFP in the diabetic rats, especially in the insulin-treated euglycemic diabetics, but fails to show significant differences. According to these similar findings, we propose that MS-CXApEn could be a sensitive parameter to assess the CAN and effects of sugar control on CAN in early diabetes.

Impaired baroreflexes have been observed in patients with diabetes. However, intensive control of blood sugar could not improve BRS in a clinical study [46]. In this study, the CXApEn at large scales for the insulin-treated euglycemic rats approached the value of the control rats (Figure 3). These findings suggest that early baroreflex impairments are immediately restored by adequate treatment in early diabetes, and therefore early intervention is essential. There are few studies of cardiac autonomic dysfunction in early diabetes. Maeda and colleagues found impaired baroreflex in a short-term model of diabetes in male Wistar rats [24]. Early impaired baroreflex without progression was also found in patients with type 2 diabetes in a recent clinical observation, as Istenes and colleagues detected subtle hypertension in 9 of 31 patients with diabetes without clinical evidence of CAN or hypertension based on 24-h blood pressure monitoring [47]. Nevertheless, the current study failed to show similar results, possibly as cardiovascular suppression by urethane affected the sensitivity of the traditional assessments of cardiac autonomic function [48]. The Bland–Altman plots still showed good agreement between the MS-CXApEn values and traditional assessments (Figure 4). This suppression highlights the sensitivity and novelty of MS-CXApEn for assessing early autonomic dysfunction in diabetes. Therefore, this novel assessment may be useful for assessing the baroreflex function of patients with diabetes during anesthesia. 

## 5. Conclusions

We propose a novel and sensitive nonlinear method for assessing the interactions between SBP and RRIs using MS-CXApEn. Our study demonstrates that this novel parameter is more sensitive than traditional indicators, including HRV and BRS, for assessing CAN in early experimental diabetes, and suggests that MS-CXApEn could help to indicate the reversal of CAN after early interventions for hyperglycemia.

## Figures and Tables

**Table 1:** Physiological parameters of the animals under urethane anesthesia.

	Normal	DM, *n* = 8	
	*n* = 8	Hyperglycemic	Euglycemic
Body weight (g)	303.75 ± 24.83 *	265.25 ± 35.90	265.25 ± 35.90
Blood sugar (mg/dL)	104.25 ± 10.98 *	360.63 ± 63.51 ^#^	183.13 ± 19.74
SBP (mmHg)	154.85 ± 16.44 *	134.53 ± 23.62	151.18 ± 17.15
DBP (mmHg)	103.79 ± 20.32 *	80.83 ± 25.27	87.85 ± 13.14
MAP (mmHg)	120.81 ± 17.90 *	98.85 ± 24.61	108.12 ± 12.96
HR (beats)	369.38 ± 34.12 *	338.13 ± 26.71	346.63 ± 19.68

Values are expressed as mean ± SD. HR: heart rate, SBP: systolic blood pressure, DBP: diastolic blood pressure, MAP: mean artery pressure ([SBP + DBP × 2]/3). * *p* < 0.05, between the normal controls and hyperglycemic diabetic rats, # *p* < 0.05 between the diabetic rats in hyperglycemic and euglycemic status after insulin injection. Significances of difference were determined as *p* < 0.05 by ANOVA with Fisher’s least significant difference post hoc test.

**Table 2:** Assessments of cardiovascular autonomic functions of the control rats, and diabetic rats in hyperglycemic and euglycemic states under urethane anesthesia.

	Normal	DM *n* = 8	
	*n* = 8	Hyperglycemic	Euglycemic
BRS_spn_ (ms/mmHg)	0.82 ± 0.58	1.04 ± 0.94	1.40 ± 1.52
BRS_phe_ (ms/mmHg)	0.82 ± 0.50	0.77 ± 0.67	1.03 ± 1.14
LFP	0.29 ± 0.19 *	0.84 ± 0.64	0.63 ± 0.42
HFP	1.55 ± 0.74	2.57 ± 1.90	3.60 ± 3.07
LF/HF	0.20 ± 0.15 *	0.35 ± 0.10 ^#^	0.21 ± 0.12
SSR	0.88 ± 0.08	0.85 ± 0.06	0.86 ± 0.07
MS-CXApEn_small_	1.23 ± 0.24 *	1.62 ± 0.13	1.51 ± 0.30
MS-CXApEn_medium_	1.01 ± 0.28 *	1.29 ± 0.12	1.21 ± 0.24
MS-CXApEn_large_	0.91 ± 0.27 *	1.17 ± 0.12	1.01 ± 0.14

BRS_spn_: spontaneous baroreflex sensitivity, BRS_phe_: baroreflex sensitivity assessed by intravenous injection of phenylephrine, LFP: low-frequency power by FFT, HFP: high-frequency power by FFT, LF/HF: ratio of LFP to HFP, SSR: SD1/SD2 ratio by Poincaré plotting of R-R intervals, MS-CXApEn_small_: averaged multiscale cross-approximate entropy between SBP and RRI from scales 1 to 3, MS-CXApEn_medium_: averaged multiscale cross-approximate entropy between SBP and RRI from scales 4 to 6, MS-CXApEn_large_: averaged multiscale cross-approximate entropy between SBP and RRI from scales 7 to 10. *: *p* < 0.05, as compared between hyperglycemic and normal rats. #: *p* < 0.05, as compared between hyperglycemic and euglycemic (insulin-treated) diabetic rats.
